# A protocol for the ERICA-ARREST feasibility study of Emergency Resuscitative Endovascular Balloon occlusion of the Aorta in Out-of-Hospital Cardiac Arrest

**DOI:** 10.1016/j.resplu.2024.100688

**Published:** 2024-06-13

**Authors:** Shadman Aziz, Jon Barratt, Noamaan Wilson-Baig, Kate Lachowycz, Rob Major, Ed B.G. Barnard, Paul Rees

**Affiliations:** a Department of Research, Audit, Innovation, and Development (RAID). East Anglian Air Ambulance, Norwich, UK; b Academic Department of Military Emergency Medicine, Royal Centre for Defence Medicine (Research & Clinical Innovation), Birmingham, UK; c Emergency Department, University Hospitals of North Midlands NHS Trust, Stoke-on-Trent, UK; d Departments of Anaesthesia and Critical Care, Wrightington, Wigan and Leigh NHS Foundation Trust, Wigan, UK; e Emergency and Urgent Care Research in Cambridge (EUReCa), PACE Section, Department of Medicine, Cambridge University, Cambridge, UK; f Academic Department of Military Medicine, Royal Centre for Defence Medicine(Research & Clinical Innovation), Birmingham, UK; g Barts Heart Centre, Barts Health NHS Trust, London, UK

**Keywords:** Out-of-hospital cardiac arrest, Prehospital, HEMS, REBOA, Protocol, Feasibility

## Abstract

**Background:**

Fewer than one in ten out-of-hospital cardiac arrest (OHCA) patients survive to hospital discharge in the UK. For prehospital teams to improve outcomes in patients who remain in refractory OHCA despite advanced life support (ALS); novel strategies that increase the likelihood of return of spontaneous circulation, whilst preserving cerebral circulation, should be investigated. Resuscitative Endovascular Balloon Occlusion of the Aorta (REBOA) has been shown to improve coronary and cerebral perfusion during cardiopulmonary resuscitation. Early, prehospital initiation of REBOA may improve outcomes in patients who do not respond to standard ALS. However, there are significant clinical, technical, and logistical challenges with rapidly delivering prehospital REBOA in OHCA; and the feasibility of delivering this intervention in the UK urban–rural setting has not been evaluated.

**Methods:**

The Emergency Resuscitative Endovascular Balloon Occlusion of the Aorta in Out-of-Hospital Cardiac Arrest (ERICA-ARREST) study is a prospective, single-arm, interventional feasibility study. The trial will enrol 20 adult patients with non-traumatic OHCA. The primary objective is to assess the feasibility of performing Zone I (supra-coeliac) aortic occlusion in patients who remain in OHCA despite standard ALS in the UK prehospital setting. The trial’s secondary objectives are to describe the hemodynamic and physiological responses to aortic occlusion; to report key time intervals; and to document adverse events when performing REBOA in this context.

**Discussion:**

Using compressed geography, and targeted dispatch, alongside a well-established femoral arterial access programme, the ERICA-ARREST study will assess the feasibility of deploying REBOA in OHCA in a mixed UK urban and rural setting.

Trial registration.

ClinicalTrials.gov (NCT06071910), registration date October 10, 2023, https://classic.clinicaltrials.gov/ct2/show/NCT06071910

## Background

Resuscitation is attempted by prehospital emergency medical services (EMS) in approximately 30,000 out-of-hospital cardiac arrest (OHCA) patients each year in the UK. However, fewer than one in ten patients (9%) survive to hospital discharge.^1^ This is significantly lower than several other countries with developed EMS systems, that report survival rates of up to 18%.^1^, ^2^ Therefore, there is considerable potential to improve outcomes from OHCA in the UK.

Return of spontaneous circulation (ROSC) is the first step to long-term survival, but is currently only achieved in 40% of OHCA patients.^1^ Furthermore, limiting irreversible ischaemic damage to the brain during ‘no-flow’ and ‘low-flow’ intervals is key to improving neurological outcomes.^3^ Recent UK public health initiatives have focused on education around bystander cardiopulmonary resuscitation (CPR) and provision of public access defibrillators.^4^ However, for OHCA patients these predetermined community factors are no longer modifiable; and once prehospital teams arrive on-scene, treatments follow advanced life support (ALS) algorithms, which have largely remained unchanged for decades.^5^ To improve outcomes in patients with refractory OHCA, novel strategies aimed at increasing the chance of ROSC, whilst preserving cerebral perfusion, should be investigated.

Resuscitative Endovascular Balloon Occlusion of the Aorta (REBOA) provides temporary occlusion of the descending aorta, and is a well-established intervention in trauma patients with torso haemorrhage.^6^, ^7^ Recently, Zone I REBOA (balloon occlusion between the origin of the left subclavian artery and the coeliac axis) has been proposed as an additional treatment for non-traumatic cardiac arrest, by mechanically increasing afterload in order to augment coronary and cerebral perfusion during CPR.^8^, ^9^

During cardiac arrest, antegrade coronary perfusion occurs exclusively in the relaxation phase of chest compression CPR^10^, ^11^, ^12^ This blood flow is driven by the diastolic pressure gradient between the proximal aorta and right atrium, developing a resultant coronary perfusion pressure (CPP).^13^ Multiple preclinical studies have demonstrated that balloon occlusion of the aorta during external chest compressions can augment CPP by increasing aortic diastolic blood pressure (AoDBP); and increase the chances of successfully achieving ROSC.^14^, ^15^, ^16^, ^17^, ^18^ Furthermore, several studies have demonstrated REBOA during CPR increases carotid blood flow and cerebral perfusion pressure,^17^, ^19^, ^20^, ^21^ and that REBOA infers greater carotid blood flow compared to bolus dose epinephrine – potentially avoiding the deleterious effects of epinephrine on cerebral perfusion.^15^, ^21^

### Rationale

Human data concerning the potential role of aortic occlusion in refractory cardiac arrest are mainly comprised of a number of case reports and observational case series.^22^, ^23^, ^24^, ^25^, ^26^, ^27^, ^28^ Therefore, a prospective evaluation of whether REBOA can improve ROSC and neurological outcomes is still required, and several randomised controlled trials of REBOA in OHCA are currently ongoing.^29^, ^30^

At the time of publication, prehospital REBOA is available in one urban UK Helicopter Emergency Medical Service (HEMS) for the management of subdiaphragmatic non-compressible torso haemorrhage.^6^ However, no UK prehospital services currently deploy REBOA in the setting of OHCA. A recent single-centre observational study has demonstrated the feasibility of prehospital REBOA in non-traumatic OHCA in Norway.^9^ However, primarily due to geographical constraints, the mean time (standard deviation) from dispatch to aortic occlusion was 45.6 (±6.3) minutes. REBOA is likely to be a time-critical intervention, and its potential benefits may be more pronounced when applied earlier after the onset of OHCA. The primary objective of this study is to determine the feasibility of prehospital aortic balloon occlusion in adult patient with refractory non-traumatic cardiac arrest in a UK setting.

### Study objectives

The Emergency Resuscitative Endovascular Balloon Occlusion of the Aorta in Out-of-Hospital Cardiac Arrest (ERICA-ARREST) study (ClinicalTrials.gov: NCT06071910) will evaluate the feasibility of deploying REBOA in a UK Helicopter Emergency Medical Service (HEMS). Using compressed geography, and targeted dispatch, alongside a well-established and governed arterial access programme (SPEAR),^31^ the study will assess the feasibility of delivering the intervention in a mixed UK urban and rural setting, within a more rapid timescale. Finally, this study aims to document the detailed physiological response to REBOA during ongoing CPR, using invasive blood pressure (IBP) monitoring, end-tidal CO2 (ETCO2) and measurement of cerebral regional oxygen saturation (rSO2) using near-infrared spectroscopy (NIRS) (See Table 1).

**Table 1:** Study outcome measures.

**Primary outcome measures**
1. Device delivery [Time frame: within 1 h of initiation]
The proportion of patients where prehospital Zone 1 REBOA for OHCA is achieved, with the balloon inserted to 35–55 cm, proximal arterial blood pressure transduced and evidence of loss of distal invasive blood pressure trace during CPR confirming aortic occlusion.
2. Procedural timings [time frame: within 1 h of initiation]
• Time taken to achieve REBOA for OHCA in minutes
• Time taken from start of the procedure (arterial catheterization) to balloon inflation in minutes
• Total duration of REBOA (inflation to final deflation) in minutes
• Time from arrival at scene to REBOA (balloon inflation) in minutes
• Time from 999 call to REBOA (balloon inflation) in minutes

**Secondary outcome measures**

1. Haemodynamic and oxygenation responses [time frame: within 1 h of initiation]
• Change in aortic diastolic pressure in mmHg
• Change in central venous pressure in mmHg
• Change in calculated coronary perfusion pressure (where cannulated) (CPP) mmHg
These hemodynamic end-points will be presented as a consecutive case series (continuous data plots per patient) and a descriptive summary for the whole cohort of absolute values and change in values between key time intervals: pre-occlusion, during occlusion, post-occlusion.
2. Near infra-red spectrometry [time frame: within 1 h of initiation]
• Change in brain regional oxygen saturation (rSO2) in %
These cerebral oxygenation end-points will be presented as a consecutive case series (continuous data plots per patient) and a descriptive summary for the whole cohort of absolute values and change in values between key time intervals: pre-occlusion, during occlusion, post-occlusion.
3. End Tidal CO2 [time frame: within 1 h of initiation]
• Change in ETCO2 in kPa
These circulatory-surrogate end-points will be presented as a consecutive case series (continuous data plots per patient) and a descriptive summary for the whole cohort of absolute values and change in values between key time intervals: pre-occlusion, during occlusion, post-occlusion.

## Methods

### Study setting

East Anglian Air Ambulance (EAAA) is a UK HEMS organisation that provides prehospital critical care in the East of England in support of the East of England Ambulance Service NHS Trust (EEAST)). The East of England is a geographic area of 20,000 km^2^ and has approximately 6.4 million inhabitants.^32^ Two or three-person HEMS teams, each with at least one physician and critical care paramedic, are dispatched from one of two bases (Cambridge and Norwich) by either helicopter or ground response vehicle, depending on patient location, weather, and time of day.^33^, ^34^ Prehospital consultants are available 24 h a day as part of the HEMS team or on call via telephone for advice.

Each year EAAA is tasked to approximately 3000 primary missions and attends 2000 patients, of which approximately 25% are OHCA. In addition to ALS interventions, the HEMS team can deliver enhanced decision-making and critical care interventions, including point-of-care ultrasonography, arterial blood pressure monitoring, central venous access, targeted vasoactive medications, prehospital emergency anaesthesia, and mechanical ventilation.^5^, ^35^

### Trial design

This is a prospective, single-arm, interventional feasibility study, conforming to Stage 2A of the IDEAL clinical trial guidelines for evaluation of a surgical intervention.^36^ The study is designed to gather information on the feasibility of the intervention, so will not include a control group for comparison. During the study recruitment period, the EAAA team will continue to attend and manage patients in the East of England. In cases of OHCA, alongside standard resuscitation, the HEMS team will identify patients who meet study eligibility criteria (Table 2).

**Table 2:** Eligibility criteria for the ERICA-ARREST feasibility trial of prehospital Zone I (supra-coeliac) REBOA for refractory non-traumatic OHCA.

**Inclusion criteria**
• Non-traumatic OHCA • 18 to 80 years old • No flow interval known or estimated to be <10 min • In cardiac arrest (with no sustained ROSC) on arrival of the EAAA ERICA-ARREST team
**Exclusion criteria**

• Patients < 18 or > 80 years of age • Known terminal illness • Severe comorbidity/multimorbidity • Inability to deploy mechanical CPR • Pregnancy (obvious or suspected)

*Abbreviations:* REBOA: resuscitative endovascular balloon occlusion of the aorta; OHCA: out-of-hospital cardiac arrest; EAAA: East Anglian Air Ambulance; CPR: cardiopulmonary resuscitation; ROSC: return of spontaneous circulation.

### Intervention

The study intervention is Zone I aortic occlusion using REBOA in patients who remain in cardiac arrest despite standard ALS.

The use of percutaneous, ultrasound-guided femoral arterial and central venous access during ongoing cardiac arrest is already well-established at EAAA. This vascular access programme is termed Specialist Percutaneous Emergency Aortic Resuscitation (SPEAR).^31^ ERICA-ARREST represents an extension of the SPEAR technique, including upsizing of the femoral arterial sheath and insertion of the Zone I REBOA catheter. Delivery of REBOA will only be conducted by teams with ERICA-ARREST-trained senior physicians in prehospital emergency medicine with extensive experience in SPEAR or other endovascular resuscitation techniques.

After arriving at an ongoing resuscitation, the HEMS team will conduct a rapid assessment to understand the circumstances of the arrest; and to ensure that high-quality CPR and ALS are underway. If eligibility criteria are met, the team will proceed to REBOA insertion following a rapid brief to other clinicians present. Both groins will be widely exposed, prepared with 2% chlorhexidine gluconate/70% isopropyl alcohol, and covered with a sterile drape.

Percutaneous cannulation of the femoral artery will be performed under ultrasound guidance. Once the access needle is in the vessel lumen, a flexible guidewire will be inserted, and the position confirmed using a long-axis ultrasound ‘wire-sweep.’ Ultrasound images of needle insertion and the guidewire position will be recorded and stored in a secure online data environment. After the guidewire has been inserted, a 5Fr sheath (MERIT Prelude®, Merit Medical, Utah, USA) will be placed. During the vascular access phase, the operator will also gain femoral venous access using another 5F sheath, which is a standard SPEAR intervention.^31^ Whilst not mandated for the study, it is encouraged as reliable central venous access is desirable for administration of vasoactive medications and enables estimation of CPP.

**Fig. 1:**
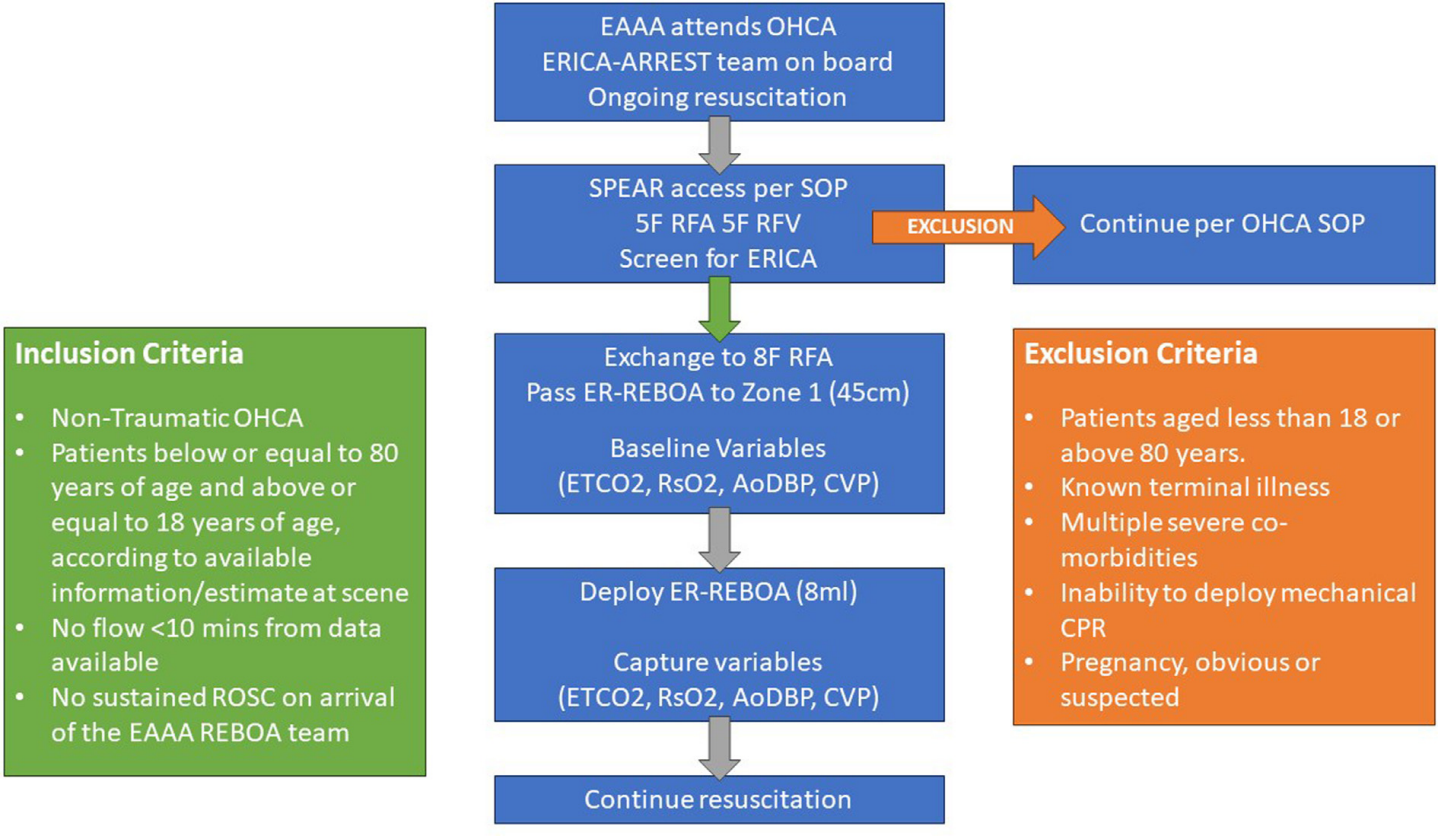
**Study flowchart.***Abbreviations*: EAAA: East Anglian Air Ambulance; OHCA: out-of-hospital cardiac arrest; SPEAR: specialist percutaneous emergency aortic resuscitation; SOP: standard operating procedure; RFA: right femoral artery; RFV: right femoral vein; ERICA-ARREST: The Emergency Resuscitative Endovascular Balloon Occlusion of the Aorta in Out-of-Hospital Cardiac Arrest study; REBOA: resuscitative endovascular balloon occlusion of the aorta; ETCO2: end-tidal CO2; RsO2: cerebral regional oxygen saturation; AoDBP: aortic diastolic blood pressure; CVP: central venous pressure; CPR: cardiopulmonary resuscitation; ROSC: return of spontaneous circulation.

In eligible ERICA-ARREST patients, the arterial sheath will be upsized to 8Fr (MERIT Prelude®, Merit Medical, Utah, USA) (Fig. 1). After removing the guidewire, the catheter sidearm will be connected to a pressure transducer to verify arterial placement and patency. A pressure-transduced ER-REBOA catheter (Prytime Medical, Boerne, TX, USA) will then be inserted through the 8Fr sheath with an approximate insertion depth of 35–55 cm based on external landmarks (xiphisternum to femoral insertion point), with the aim of placing the balloon in Zone 1.^37^

Alongside standard ETCO2 and cerebral oximetry monitoring, the proximal and distal arterial pressures will be measured during balloon inflation. The REBOA operator will then inflate the balloon with saline until the distal arterial pressure waveform is lost. Once the balloon is inflated, the REBOA catheter will be secured to prevent migration or dislodgement. If ROSC is achieved without significant instability, the balloon will be gradually deflated by 0.5 ml every 30 s, guided by invasive physiology. Post resuscitation care will follow standard EAAA and Resuscitation Council UK guidelines.^38^ Resuscitation will be terminated if the patient remains in cardiac arrest following standard EAAA protocols; considering the clinical context, patient factors, point-of-care ultrasound findings and blood gas analysis if available.

### Timescale

The study will continue until 20 ERICA-ARREST patients have been enrolled, or 24 months have elapsed. The study will end following the death or hospital discharge of the final patient or after 90 days since the hospital admission date of the final trial patient.

### Data collection

A paper case report form (CRF) will be completed for each case by the trial clinician undertaking the procedure (supplementary appendix). Within 24 h of the procedure, a structured post-event debrief will be conducted in collaboration with the Chief Investigator (CI) to ensure compliance with the protocol and correct documentation. Additional data relating to patient demographics and outcomes will be retrieved from data collected as part of routine patient care and aftercare (Table 3).

**Table 3:** Data collected per patient for the ERICA-ARREST feasibility trial of prehospital Zone I (supra-coeliac) REBOA for refractory non-traumatic OHCA.

**Field**	**Data points collected**	**Source**
End-tidal CO2 (ETCO2)	Per 30 s intervals throughout	Zoll X-series
Aortic diastolic pressure (AoDBP)	Per 30 s intervals throughout	Zoll X-series
Central venous/femoral venous pressure if available	Per 30 s intervals throughout	Zoll X-series
Cerebral oximetry (rSO2)	Per 1 s interval throughout	Nonin co-pilot cerebral oximeter
Age (Years)		Patient record/clinical team
Sex		Patient record/clinical team
Weight (Estimated kg)		Patient record/clinical team
Timings (hh:mm)	Estimated time of arrest 999 call EEAST arrival Estimated CPR time Estimated time of 1st defibrillation HEMS dispatch HEMS arrival HEMS depart HEMS handover at hospital Sustained ROSC time PLE	Patient record/clinical team
Cardiac rhythm	On EEAST arrival (& Time or rhythm) On HEMS arrival	Patient record/clinical team
Witnessed arrest	Yes/No	Patient record/clinical team
Bystander CPR	Yes/No	Patient record/clinical team
Bystander defibrillation	Yes/No	Patient record/clinical team
Suspected aetiology	Cardiac – myocardial infarction Cardiac – arrhythmia Cardiac – heart failure Respiratory – pulmonary embolism Respiratory – other Other	Patient record/clinical team
Known comorbidities		Patient record/clinical team
Number of defibrillation shocks		Patient record/clinical team
Adrenaline administered	Yes/No Total cumulative dose given	Patient record/clinical team
Airway management	Supraglottic airway Endotracheal Tube	Patient record/clinical team
Ventilation strategy	Oxylog/Manual	Patient record/clinical team
Survival to discharge	Yes/No	Aftercare
Complications related to vascular access	e.g. Local haemorrhage, lower limb ischaemia, infection, aortic dissection, femoral vessel dissection, rupture or injury, surgical intervention/repair needed	Aftercare
In contact with Aftercare	Yes/No	Aftercare
Consent given	Yes/No	Trial team/Aftercare
Date of consent		Trial team/Aftercare
Survival to 30 days	Yes/No	Aftercare

*Abbreviations:* EEAST: East of England Ambulance Service NHS Trust; CPR: cardiopulmonary resuscitation; HEMS: Helicopter Emergency Medical Service; ROSC: return of spontaneous circulation; PLE: pronounced life extinct.

At EAAA, there is a dedicated team of Aftercare nurses and paramedics who routinely contact patients and families (including bereaved families), to ensure they are receiving the appropriate support following their incident. The Aftercare team are able to signpost individuals to the appropriate services for physical, emotional, financial and/or legal support if these needs are identified. Furthermore, they are able to arrange meetings between the patient/family and the clinical team if they have any questions about the care they received. Patient outcome data is routinely collected during the aftercare process. The Aftercare team will also facilitate the process for obtaining consent.

### Data management and confidentiality

Each patient will be given a unique study number as an identifier. Electronic data for analysis will not contain identifiable data and will be stored securely in password-protected files in a secure data environment. All paper files will be stored in locked cabinets in a restricted access office at EAAA study sites (Norwich and Cambridge). Data access will only be granted by the CI to authorised trial team members.

### Statistical methods

Descriptive statistics will be calculated to describe the processes and physiological endpoints, including technical, clinical, and safety endpoints. Computed statistics will be appropriate to the normality of the data and presented as proportions (±95% confidence intervals), means (±standard deviation) and medians (±interquartile range) as appropriate. Data will be processed and analysed using a combination of Excel (Microsoft, Redmond, WA, USA) and the R statistical programming language (R Studio, R Foundation for Statistical Computing, Vienna, Austria).

## Ethics and funding

### Research ethics approval

The London-Bromley REC has approved the trial (23/LO/0996). The trial is registered at ClinicalTrials.gov (NCT06071910).

### Patient and public involvement

The EAAA patient forum group were consulted during the development phase of the study. This is a diverse group of patients and relatives, including survivors of cardiac arrest, as well as patients treated for other conditions such as major trauma. A facilitated discussion was held on May 16, 2023 to gather views on the study proposal. Feedback from participants was considered in the design of the project. In particular, the patient forum strongly supported the study’s consent and information-sharing processes. The patient forum will continue to be consulted and updated on the trial during quarterly meetings, and the final results will be presented to the group following the conclusion of the trial.

### Patient consent

Patients eligible for the study will be in cardiac arrest and, therefore, unable to give consent to enter the study. Furthermore, given the time-critical nature of OHCA, there will not be sufficient time to consult the patient’s next-of-kin or a legally authorised consultee in order for them to make an informed decision about the patient’s involvement in the study. In England, the legal framework for conducting non-CTIMP trials in this context follows the Mental Capacity Act 2005 principles.^39^ Using this legal framework, precedent from previous trials of OHCA in England, and approval from the NHS Health Research Authority REC, the initial study enrolment will take place without consent.^39^, ^40^ Subsequent written informed consent will be sought from patients who regain capacity or a legally authorised consultee if the patient lacks capacity. Consent will only relate to continuation in the study and future follow-up, as the intervention will have already occurred. This subsequent informed consent process will run alongside routine contact and support from the EAAA Aftercare team. For patients who die before consent can be obtained, in a process agreed with the REC, the next-of-kin will receive a letter informing them that the patient was recruited into the study. This will also be followed up with routine contact from the EAAA Aftercare team, with a member of the research team additionally available to answer any questions related to the study.

### Research governance

A trial management committee (TMC) has been assembled to conduct the day-to-day administration of the study. The TMC consists of an independent chair and key members from the study team (CI, PI, study coordinator and data manager). The TMC will monitor all aspects of the conduct and progress of the trial, including adherence to the protocol, Good Clinical Practice; and reporting of any adverse events (AE) or serious adverse events (SAE) to the sponsor (Queen Mary University of London) and Research Ethics Committee (REC).

AEs are defined as any untoward medical occurrence in a participant to whom an intervention has been administered. SAEs are defined as any adverse event that is life-threatening, requires hospitalisation or prolongation of current hospitalisation or results in persistent or significant disability or incapacity. However, as this study recruits patients who are in refractory OHCA; untoward medical occurrences, death, prolonged hospitalisation, and disability are common, expected and often inevitable outcomes. Therefore, these events will not be recorded as AEs/SAEs unless considered to be ‘unexpected’ or directly related to the intervention.

A trial steering committee (TSC) has also been assembled with an independent chair, three independent experts in the field of adult resuscitation medicine, and a patient and public representative. The TSC will meet before patient recruitment, at 3-months and 6-months after the trial has started and then every 6-months thereafter. The TSC will make recommendations after considering the latest external evidence, safety, feasibility and any other arising issues.

Substantial amendments to the current protocol (v1.0, October 30, 2023) will only occur if approved by the REC. The protocol is available on the trial website: https://www.eaaa.org.uk/our-work/clinical-research/erica.

### Funding

The research costs of the study are part-funded by the Rosetrees Trust, with reimbursement of excess treatment costs from the Department of Health & Social Care and study support costs from the East of England Clinical Research Network (National Institute of Health Research). Donations towards development and set-up of the study were also received from the Dowager Countess Elanor Peel Trust (DCEPT), The Charles Wolfson Charitable Trust, The Thriplow Charitable Trust, and The Helen Roll Charity. The funding bodies will be acknowledged in publications but will not approve results or publications.

### Dissemination of findings

The trial results will be presented locally, regionally, and nationally at relevant academic conferences. A manuscript summarising the study’s findings will be written by the working group following completion for consideration of publication in a peer-reviewed medical journal.

## Conclusion

The ERICA-ARREST study will evaluate the feasibility of deploying REBOA in OHCA for the first time in a UK Helicopter Emergency Medical Service. Using compressed geography, and targeted dispatch, alongside a well-established femoral arterial access programme, the study will assess the feasibility of delivering the intervention in a mixed UK urban and rural setting, within a rapid timescale. The results of this trial will inform the design of future efficacy studies, which will investigate whether the implementation of REBOA can improve survival in OHCA patients who do not respond to standard ALS.

## CRediT authorship contribution statement

**Shadman Aziz:** Writing – original draft, Visualization, Project administration. **Jon Barratt:** Writing – review & editing, Project administration, Methodology. **Noamaan Wilson-Baig:** Writing – review & editing, Project administration, Methodology. **Kate Lachowycz:** Writing – review & editing, Resources, Project administration, Methodology, Funding acquisition, Formal analysis. **Rob Major:** Writing – review & editing, Resources, Project administration, Methodology, Funding acquisition. **Ed B.G. Barnard:** Writing – review & editing, Project administration, Methodology. **Paul Rees:** Writing – review & editing, Supervision, Project administration, Methodology, Funding acquisition, Formal analysis.

## Declaration of competing interest

The authors declare that they have no known competing financial interests or personal relationships that could have appeared to influence the work reported in this paper.
