# Supplementary material for: A protocol for the ERICA-ARREST feasibility study of Emergency Resuscitative Endovascular Balloon occlusion of the Aorta in Out-of-Hospital Cardiac Arrest
Source: Resusc Plus. 2024 Jun 13;19:100688. doi: 10.1016/j.resplu.2024.100688 (PMC11225899; doi:10.1016/j.resplu.2024.100688)
Supplement: Supplementary Data 3 [file mmc3.docx]

**ERICA-ARREST Case Report Form**

| **Date** |  | **Completed by** |  |
| --- | --- | --- | --- |

|  | | | **Yes** | **No** |
| --- | --- | --- | --- | --- |
| REBOA achieved? | | |  |  |
| ERICA checklist completed prior to enrolment | | |  |  |
| Was there palpable pulse in the radial artery after balloon occlusion | | |  |  |
| Did the procedure interrupt the CPR? | | |  |  |
| Ultrasound image recorded | | |  |  |
| Cannulation site |  | Number of attempts |  | |

| **Key timings** | **hh:mm** |
| --- | --- |
| Arrival at patient |  |
| Enrolment time (when checklist completed) |  |
| Time of balloon occlusion (when confirmed R1 pulsatile pressure on Lucas and R2 zero) |  |
| Time(s) of ROSC |  |
|  |  |
|  |  |
| Time of deflation start |  |
| Time of deflation complete |  |
| Departure from scene |  |
| Handover to hospital |  |
| PLE time |  |

| **Factors influencing procedure** | **Easy / Difficult / Impossible** |
| --- | --- |
| **Technical and clinical factors** | |
| Body habitus |  |
| Ease of cannulation |  |
| Ease of insertion of guidewire |  |
| Ease of insertion of introducer |  |
| Ease of REBOA insertion |  |
| **Environmental factors** | |
| Weather |  |
| Temperature |  |
| Light conditions |  |
| Space and access |  |

**ERICA-ARREST Case Report Form**

| **Complications / adverse events NB: Include timings where relevant** |
| --- |
|  |
| **Physician comments** |
|  |
