# Supplementary material for: A protocol for the ERICA-ARREST feasibility study of Emergency Resuscitative Endovascular Balloon occlusion of the Aorta in Out-of-Hospital Cardiac Arrest
Source: Resusc Plus. 2024 Jun 13;19:100688. doi: 10.1016/j.resplu.2024.100688 (PMC11225899; doi:10.1016/j.resplu.2024.100688)
Supplement: Supplementary Data 4 [file mmc4.docx]

**Participant Information Sheet**

**E**mergency **R**esuscitative Endovascular Balloon Occlusion of the Aorta **i**n out of hospital **c**ardiac **a**rrest (**ERICA-ARREST**) Study

IRAS: 296654

REC: TBC

Chief Investigator: Dr Paul Rees

Before you were admitted to hospital, you were enrolled in a clinical research study called the ERICA-ARREST Study by East Anglian Air Ambulance (EAAA). The necessity of immediate treatment meant that we could not take your consent, or ask a relative or friend, at the time. We are now seeking your consent to continue to take part in the research study, which will involve us keeping some information we already hold about your care. It is important for you to understand why this research is being done and what it means for you. Please take time to read the following information leaflet carefully and decide whether or not you would like to continue to take part. Please feel free to contact the research team at any point if there is anything that is not clear, or if you need more information. Our team will answer any questions you have.

**Explanation of the ERICA Study**

Sudden out-of-hospital cardiac arrest is the most common mission for the East Anglian Air Ambulance (EAAA). Around one in five of our cardiac arrest patients survive, but across the country the rate of survival is below 10% - this condition needs new treatments to help improve survival. . The current management of out-of-hospital cardiac arrest (OHCA) includes cardiopulmonary resuscitation (CPR) to supply blood to the heart and brain. Optimising blood flow to the heart and brain is important for survival. However even during well-performed CPR, blood flow to the brain and heart is well below normal levels.

In animal studies putting a balloon into a large artery, close to the heart , via the groin, showed an improvement in heart and brain blood flow, and short-term survival from cardiac arrest. Reports from human studies have shown similar effects in patients who suffer from cardiac arrest.

EAAA has clinicians highly trained and experienced in resuscitation for cardiac arrest, andnd inserting medical tubes into patients' arteries and veins. We are undertaking a ‘first in the UK’ research study, investigating the effectiveness of placing a balloon catheter into the aorta in OHCA patients, who are undergoing resuscitation (CPR). This project is supported by, and undertaken in partnership with, Queen Mary University of London..

**Why was I chosen?**

You were chosen because you suffered a cardiac arrest. At this time, the doctor who was treating you checked carefully that you were suitable to take part in this study. As a result, you will have been treated with REBOA during your initial resuscitation and emergency care.

**What does taking part involve?**

The aim of the research is to see if putting the balloon catheter in 20 carefully selected patients, who have suffered a cardiac arrest and are being resuscitated by the ambulance service is feasible. The study looks at the effectiveness of putting the balloon into patients, in cardiac arrest, in the pre-hospital setting. Selected patients undergoing resuscitation have had this balloon inserted at the scene of the incident by EAAA clinicians. You are receiving this as you are one of the 20 patients recruited into the trail who had a balloon inserted during your cardiac arrest.

During the procedure being studied, full active resuscitation will continue to be provided by our EAAA crew and the ambulance service resuscitation teams, and others. The delivery of the procedure will not have adversely affected the resuscitation in any way – all the usual emergency procedures and medication treatments will continue as usual, in an attempt to restart the heart.

The balloon was inserted into an artery in your groin and threaded into a larger artery close to your heart (the aorta). A small balloon was inflated which stopped blood flowing into your abdomen for a short period of time. It is a well-established technique in medical resuscitation, also known as REBOA (**R**esuscitative **E**ndovascular **B**alloon **O**cclusion of the **A**orta). It is our hope thathe balloon increased the blood supply to your heart and brain during your resuscitation.


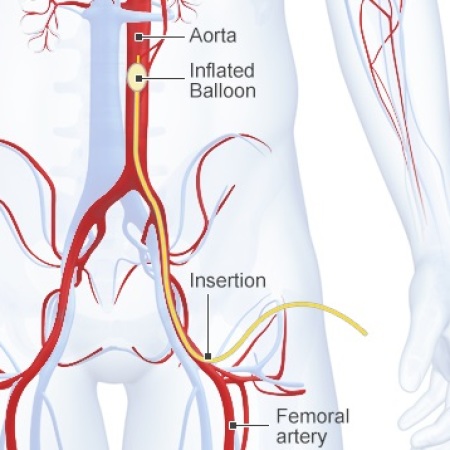


You were then taken to hospital while receiving standard critical care from the EAAA team.As soon as your heart was restarted,the balloon was removed from your body. While the balloon was inflated EAAA carefully monitored the balloon and your blood pressure, and took blood pressure readings which we wish to use for research into better resuscitation techniques for cardiac arrest patients.

**What are the possible benefits of taking part?**We are researching the benefits of this balloon technique, which could possibly make restarting the heart of cardiac arrest patients easier and giving them a better chance of recovery. If we can demonstrate this technique is feasible, we could use it on other patients to see if it improves overall survival rates from cardiac arrest.

**Who is organising and paying for the study?**

This study is sponsored by Queen Mary University of London. The study is funded by EAAA, with support from the Rosetrees Trust. The research is being carried out by a group of experienced researchers from Queen Mary University of London and EAAA.

**What are the possible disadvantages and risks of taking part?**

The risks from you continuing in the study are negligible at this point. There is a very small risk (less than 1 in 300) that we may have damaged the blood vessel in your groin as we inserted the balloon catheter. We monitor this carefully and the hospital you were taken to also watches carefully for any complications and is able to treat any potential complications. All complications and safety concerns are fed back to the research team.

**What will happen to my information/data?**

Any information we hold about you is anonymised and securely stored as part of the research study. Once the trial is completed, we aim to publish our findings in a peer reviewed medical journal to show other medical professionals the results. We hope this will add to and stimulate further research into improving survival from OHCA.

**Who will know I am taking part in the study?**

All the information we collect during the research will be kept confidential and there are strict laws which safeguard the privacy of the patient at every stage. Only certain members of the research team will have access to your information in order to contact you, if required.

It is a requirement that your records in this study, together with any relevant medical records, are made available if requested by monitors from the sponsors, and the Regulatory Authorities whose roles it is to check this research is properly conducted and the interests of those taking part in this study are protected. All these individuals are under a duty to keep your records confidential.

**Can I say no/still withdraw from the study?**

Yes. You have already had the balloon procedure undertaken by EAAA when you were unconscious during your cardiac arrest. If you wish you can still withdraw from this research study. This may mean that we are unable to use your data.

While you were still recovering in hospital and unable to give consent, we may have asked a consultee (such as a partner, family member, friend/carer or a professional such as a doctor) to advise on your behalf. They would have been given information about the study and signed a declaration form. Now that you are able to make a decision, it is up to you to decide whether to continue to take part in the study.

**How will we use information about you?**

We will need to use information from your medical records for this research project.

This information will include your name, NHS number and contact details. People will use this information to do the research or to check your records to make sure that the research is being done properly. People who do not need to know who you are will not be able to see your name or contact details. Your data will have a code number instead.

We will keep all information about you safe and secure. We will write our reports in a way that no-one can work out that you took part in the study. Once we have finished the study, we will keep some of the data so we can check the results. Anonymised information may be shared with other researchers to advance clinical knowledge and benefit patients in the future. It will not be possible to identify you personally from any information shared.

**What are your choices about how your information is used?**

- You can stop being part of the study at any time, without giving a reason, but we will keep information about you that we already have. Holding this information allows us to make contact in the future in case any issues arise from the study.
- We need to manage your records in specific ways for the research to be reliable. This means that we won’t be able to let you see or change the data we hold about you.

**Where can you find out more about how your information is used?**

You can find out more about how we use your information:

- at [www.hra.nhs.uk/information-about-patients/](https://www.hra.nhs.uk/information-about-patients/)
- viewing EAAA’s patient privacy notice at: [www.eaaa.org.uk/our-work/clinical-research](http://www.eaaa.org.uk/our-work/clinical-research)
- by asking one of the research team
- by sending an email to [ERICA@eaaa.org.uk](mailto:ERICA@eaaa.org.uk) or the sponsor’s data protection officer [data-protection@qmul.ac.uk](mailto:data-protection@qmul.ac.uk)
- by ringing EAAA on 03450 669 999

Thank you for taking the time to read this information.

Contacts:

EAAA Aftercare team: [Aftercare@EAAA.org.uk](mailto:Aftercare@EAAA.org.uk)

ERICA research team: [ERICA@eaaa.org.uk](mailto:ERICA@eaaa.org.uk)

Chief Investigator: Paul.Rees@eaaa.org.uk
