# Supplementary material for: A protocol for the ERICA-ARREST feasibility study of Emergency Resuscitative Endovascular Balloon occlusion of the Aorta in Out-of-Hospital Cardiac Arrest
Source: Resusc Plus. 2024 Jun 13;19:100688. doi: 10.1016/j.resplu.2024.100688 (PMC11225899; doi:10.1016/j.resplu.2024.100688)
Supplement: Supplementary Data 5 [file mmc5.docx]

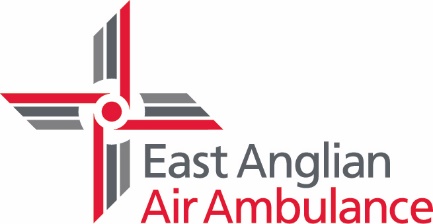

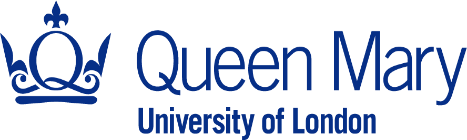


IRAS ID: 296654

Study Number:

Participant Identification Number for this trial:

**CONSENT FORM**

**Name of study: E**mergency **R**esuscitative Endovascular Balloon Occlusion of the Aorta **i**n out of hospital **c**ardiac **a**rrest (ERICA-ARREST) Study

**Name of Researcher:**

Please initial box

1. I confirm that I have read the information sheet dated.................... (version............) for the
   above study. I have had the opportunity to consider the information, ask questions and have
   had these answered satisfactorily.
2. I understand that my participation is voluntary and that I am free to withdraw at any time
   without giving any reason, without my medical care or legal rights being affected.
3. I understand that relevant sections of my medical notes and data collected during
   the study, may be looked at by individuals from East Anglian Air Ambulace, from regulatory
   authorities or from Queen Mary University of London, where it is relevant to my taking part
   in this research. I give permission for these individuals to have access to my records.
4. I understand that the information collected about me will be used to support
   other research in the future and may be shared anonymously with other researchers.
5. I understand that the information held and maintained by East Anglian Air Ambulance may
   be used to help contact me or provide information about my health status.
6. I agree to take part in the above study.

Name of Participant Date Signature

Name of Person taking consent Date Signature
