# Supplementary material for: A protocol for the ERICA-ARREST feasibility study of Emergency Resuscitative Endovascular Balloon occlusion of the Aorta in Out-of-Hospital Cardiac Arrest
Source: Resusc Plus. 2024 Jun 13;19:100688. doi: 10.1016/j.resplu.2024.100688 (PMC11225899; doi:10.1016/j.resplu.2024.100688)
Supplement: Supplementary Data 6 [file mmc6.docx]

| Data category | Information |
| --- | --- |
| Primary registry and trial identifying number | ClinicalTrials.gov  NCT06071910 |
| Date of registration in primary registry | 14/07/2023 |
| Secondary identifying numbers |  |
| Source(s) of monetary or material support | The research costs of the study are part-funded by the Rosetrees Trust, with reimbursement of excess treatment costs from the Department of Health & Social Care and study support costs from the East of England Clinical Research Network (National Institute of Health Research). Donations towards development and set-up of the study were also received from the Dowager Countess Elanor Peel Trust (DCEPT), The Charles Wolfson Charitable Trust, The Thriplow Charitable Trust, and The Helen Roll Charity. |
| Primary sponsor | Queen Mary University of London  Contact: Mays Jawad, Research Governance Operations Manager,  m.jawad@qmul.ac.uk |
| Secondary sponsor(s) |  |
| Contact for public queries | Dr Paul Rees  paul.rees@eaaa.org.uk |
| Contact for scientific queries | Dr Kate Lachowycz  kate.lachowycz@eaaa.org.uk |
| Public title | Emergency Resuscitative Endovascular Balloon Occlusion of the Aorta in Out of Hospital Cardiac Arrest |
| Scientific title | Emergency Resuscitative Endovascular Balloon Occlusion of the Aorta in Out of Hospital Cardiac Arrest |
| Countries of recruitment | United Kingdom |
| Health condition(s) or problem(s) studied | Out-of-hospital cardiac arrest |
| Intervention(s) | Delivery of ER-REBOA catheter to achieve aortic occlusion during resuscitation for out of hospital cardiac arrest, refractory to conventional advanced life support. |
|  | Device: ER-REBOA catheter |
| Key inclusion and exclusion criteria | Non-traumatic OHCA |
|  | 18 to 80 years old |
|  | No flow interval known or estimated to be <10 minutes |
|  | In cardiac arrest (with no sustained ROSC) on arrival of the EAAA ERICA-ARREST team |
| Study type | Prospective, single-arm, interventional feasibility study, conforming to Stage 2A of the IDEAL clinical trial guidelines for evaluation of a surgical intervention |
| Date of first enrolment | 04/06/2024 |
| Target sample size | 20 |
| Recruitment status | Not yet recruiting |
| Primary outcome(s) | 1. Device delivery [Time frame: within 1 hour of initiation]  The proportion of patients where prehospital Zone 1 REBOA for OHCA is achieved, with the balloon inserted to 35 - 55 cm, proximal arterial blood pressure transduced and evidence of loss of distal invasive blood pressure trace during CPR confirming aortic occlusion.  2. Procedural timings [time frame: within 1 hour of initiation]  Time taken to achieve REBOA for OHCA in minutes  o Time taken from start of the procedure (arterial catheterization) to balloon inflation in minutes  o Total duration of REBOA (inflation to final deflation) in minutes  o Time from arrival at scene to REBOA (balloon inflation) in minutes  o Time from 999 call to REBOA (balloon inflation) in minutes |
| Key secondary outcomes | 1. Haemodynamic and oxygenation responses [time frame: within 1 hour of initiation]  Change in aortic diastolic pressure in mmHg  o Change in central venous pressure in mmHg  o Change in calculated coronary perfusion pressure (where cannulated) (CPP) mmHg  These hemodynamic end-points will be presented as a consecutive case series (continuous data plots per patient) and a descriptive summary for the whole cohort of absolute values and change in values between key time intervals: pre-occlusion, during occlusion, post-occlusion.  2. Near infra-red spectrometry [time frame: within 1 hour of initiation]  Change in brain regional oxygen saturation (rSO2) in %  These cerebral oxygenation end-points will be presented as a consecutive case series (continuous data plots per patient) and a descriptive summary for the whole cohort of absolute values and change in values between key time intervals: pre-occlusion, during occlusion, post-occlusion.  3. End Tidal CO2 [time frame: within 1 hour of initiation]  Change in ETCO2 in kPa  These circulatory-surrogate end-points will be presented as a consecutive case series (continuous data plots per patient) and a descriptive summary for the whole cohort of absolute values and change in values between key time intervals: pre-occlusion, during occlusion, post-occlusion. |
