# Supplementary material for: A protocol for the ERICA-ARREST feasibility study of Emergency Resuscitative Endovascular Balloon occlusion of the Aorta in Out-of-Hospital Cardiac Arrest
Source: Resusc Plus. 2024 Jun 13;19:100688. doi: 10.1016/j.resplu.2024.100688 (PMC11225899; doi:10.1016/j.resplu.2024.100688)
Supplement: Supplementary Data 1 [file mmc1.pdf]

NHS
Health Research
Authority

London - Bromley Research Ethics Committee
2 Redman Place
Stratford
London
E20 1JQ

Telephone: 0207 104 8118

23 January 2024

Dr Paul Rees
Reader in Military Medicine
QMUL
Blizard Institute, The Faculty of Medicine and Dentistry
Queen Mary University of London
The Blizard Building, 4 Newark Street, London
E1 2AT

Dear Dr Rees,

**Study title:**	**Emergency Resuscitative Endovascular Balloon Occlusion of the Aorta in out of hospital Cardiac Arrest**
**REC reference:**	**23/LO/0996**
**Protocol number:**	**ERICA 001**
**IRAS project ID:**	**296654**

Thank you for your letter responding to the Research Ethics Committee's (REC) request for further information on the above research and submitting revised documentation.

The further information has been considered on behalf of the Committee by the Chair and Lead Reviewer.

Confirmation of ethical opinion

On behalf of the Committee, I am pleased to confirm a favourable ethical opinion for the above research on the basis described in the application form, protocol and supporting documentation as revised, subject to the conditions specified below.

Mental Capacity Act 2005 (England and Wales)

I confirm that the committee has approved this research project for the purposes of the Mental Capacity Act 2005 (England and Wales). The committee is satisfied that the requirements of section 31 of the Act will be met in relation to research carried out as part of this project on, or in relation to, a person who lacks capacity to consent to taking part in the project.

Mental Capacity Act (Northern Ireland) 2016

The Committee approved this research project for the purposes of the Mental Capacity Act (Northern Ireland) 2016. The Committee is satisfied that the requirements of Part 8 of the Act will be met in relation to research carried out as part of this project on, or in relation to, a person who lacks capacity to consent to taking part in the project.

Relevance of the research to the impairing condition

The Committee agreed the research was connected with an impairing condition affecting persons lacking capacity or with the treatment of the condition.

Justification for including adults lacking capacity to meet the research objectives

The Committee agreed the research could not be carried out as effectively if it was confined to participants able to give consent.

Arrangements for appointing consultees

The Committee considered the arrangements set out in the application for appointing consultees under Section 32 of the Mental Capacity Act 2005 (England and Wales) and the equivalent Section 135 of the Mental Capacity Act (Northern Ireland) 2016) to advise on whether participants lacking capacity should take part and on what their wishes and feelings would have likely to have been if they had capacity.

After discussion the Committee agreed that reasonable arrangements were in place for appointing consultees.

The arrangements for recruitment in an emergency setting

The Committee noted that the research would take place in circumstances involving the provision of urgent treatment to participants lacking capacity.

The Committee agreed that, in the circumstances, it was justified to recruit participants prior to obtaining advice from a consultee under the provisions in Section 32(8) and (9) of the Mental Capacity Act 2005 (England and Wales) and the equivalent Section 136 of the Mental Capacity Act (Northern Ireland) 2016).

Balance between benefit and risk, burden, and intrusion

The Committee agreed that the research has the potential to benefit participants lacking capacity without imposing a disproportionate burden on them.

Additional safeguards

The Committee was satisfied that reasonable arrangements would be in place to comply with the additional safeguards set out in Section 33 of the Mental Capacity Act 2005 (England and Wales) and the equivalent Section 137 of the Mental Capacity Act (Northern Ireland) 2016).

Good practice principles and responsibilities

The UK Policy Framework for Health and Social Care Research sets out principles of good practice in the management and conduct of health and social care research. It also outlines the responsibilities of individuals and organisations, including those related to the four elements of research transparency:

1. registering research studies
2. reporting results
3. informing participants
4. sharing study data and tissue

Conditions of the favourable opinion

The REC favourable opinion is subject to the following conditions being met prior to the start of the study.

Confirmation of Capacity and Capability (in England, Northern Ireland and Wales) or NHS management permission (in Scotland) should be sought from all NHS organisations involved in the study in accordance with NHS research governance arrangements. Each NHS organisation must confirm through the signing of agreements and/or other documents that it has given permission for the research to proceed (except where explicitly specified otherwise).

Guidance on applying for HRA and HCRW Approval (England and Wales)/ NHS permission for research is available in the Integrated Research Application System.

For non-NHS sites, site management permission should be obtained in accordance with the procedures of the relevant host organisation.

Sponsors are not required to notify the Committee of management permissions from host organisations

Registration of Clinical Trials

All research should be registered in a publicly accessible database and we expect all researchers, research sponsors and others to meet this fundamental best practice standard.

It is a condition of the REC favourable opinion that **all clinical trials are registered** on a publicly accessible database within six weeks of recruiting the first research participant. For this purpose, ‘clinical trials’ are defined as:

- clinical trial of an investigational medicinal product

- clinical investigation or other study of a medical device
- combined trial of an investigational medicinal product and an investigational medical device
- other clinical trial to study a novel intervention or randomised clinical trial to compare interventions in clinical practice.

Failure to register a clinical trial is a breach of these approval conditions, unless a deferral has been agreed by the HRA (for more information on registration and requesting a deferral see: Research registration and research project identifiers).

If you have not already included registration details in your IRAS application form you should notify the REC of the registration details as soon as possible.

Publication of Your Research Summary

We will publish your research summary for the above study on the research summaries section of our website, together with your contact details, no earlier than three months from the date of this favourable opinion letter.

Should you wish to provide a substitute contact point, make a request to defer, or require further information, please visit:
https://www.hra.nhs.uk/planning-and-improving-research/application-summaries/research-summaries/

N.B. If your study is related to COVID-19 we will aim to publish your research summary within 3 days rather than three months.

During this public health emergency, it is vital that everyone can promptly identify all relevant research related to COVID-19 that is taking place globally. If you haven't already done so, please register your study on a public registry as soon as possible and provide the REC with the registration detail, which will be posted alongside other information relating to your project. We are also asking sponsors not to request deferral of publication of research summary for any projects relating to COVID-19. In addition, to facilitate finding and extracting studies related to COVID-19 from public databases, please enter the WHO official acronym for the coronavirus disease (COVID-19) in the full title of your study. Approved COVID-19 studies can be found at: https://www.hra.nhs.uk/covid-19-research/approved-covid-19-research/

It is the responsibility of the sponsor to ensure that all the conditions are complied with before the start of the study or its initiation at a particular site (as applicable).

After ethical review: Reporting requirements

The attached document "After ethical review – guidance for researchers" gives detailed guidance on reporting requirements for studies with a favourable opinion, including:

- Notifying substantial amendments
- Adding new sites and investigators

- Notification of serious breaches of the protocol
- Progress and safety reports
- Notifying the end of the study, including early termination of the study
- Final report
- Reporting results

The latest guidance on these topics can be found at https://www.hra.nhs.uk/approvals-amendments/managing-your-approval/.

Approved documents

The final list of documents reviewed and approved by the Committee is as follows:

Document	*Version*	*Date*
Covering letter on headed paper [ERICA Cover Letter]	1	24 November 2023
IRAS Application Form [IRAS_Form_01122023]		01 December 2023
Letter from funder [A2017 Funding award letter, Dr Paul Rees]		
Letter from sponsor [AD6a_Queen_Mary_Sponsorship_(with_conditions)_letter_(Interventional_and_Research)]	8.0	17 November 2023
Letters of invitation to participant [Aftercare contact letter]	1	19 December 2023
Other [Insurance Certificate Sponsor]		
Other [Insurance certificate Site]		
Other [Email are update to funding]		
Participant consent form [ERICA consent form patient]	3	16 January 2024
Participant consent form [ERICA consent form consultee]	3	16 January 2024
Participant consent form [ERICA consent form patient tracked]	3	16 January 2024
Participant consent form [ERICA consent form consultee tracked]	3	16 January 2024
Participant information sheet (PIS) [ERICA PIS]	3	16 January 2024
Participant information sheet (PIS) [ERICA Consultee IS]	3	16 January 2024
Participant information sheet (PIS) [ERICA PIS tracked]	3	16 January 2024
Participant information sheet (PIS) [ERICA Consultee IS tracked]	3	16 January 2024
Research protocol or project proposal [ERICA IRAS JRMO Protocol]	1	30 October 2023
Summary CV for Chief Investigator (CI) [CV REES]		23 November 2023

Statement of compliance

The Committee is constituted in accordance with the Governance Arrangements for Research Ethics Committees and complies fully with the Standard Operating Procedures for Research Ethics Committees in the UK.

User Feedback

The Health Research Authority is continually striving to provide a high quality service to all applicants and sponsors. You are invited to give your view of the service you have received and the application procedure. If you wish to make your views known please use the feedback form

available on the HRA website:
http://www.hra.nhs.uk/about-the-hra/governance/quality-assurance/

HRA Learning

We are pleased to welcome researchers and research staff to our HRA Learning Events and online learning opportunities– see details at:
https://www.hra.nhs.uk/planning-and-improving-research/learning/

**IRAS project ID: 296654**	**Please quote this number on all correspondence**

With the Committee's best wishes for the success of this project.

Yours sincerely

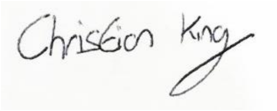

Christian King
Approvals Specialist

On behalf of

Mrs Helen Jane Wood
Chair

Email:bromley.rec@hra.nhs.uk

Enclosures: "After ethical review – guidance for researchers"

Copy to: Dr Mays Jawad, Sponsor's Representative

Lead Nation
approvals@hra.nhs.uk
